# The History and Capabilities of Herbal Simples

**Published:** 1890-01-11

**Authors:** W. T. Fernie


### The History and Capabilities of Herbal Simples.

By W. T. Fernie, M.D.

### III.—The Wood-Anemone, Angelica, Aniseed.

The wood-anemone, with its lovely pink-white petals and drooping blossoms, is one of our best-known and most beautiful spring flowers. Botanists do not now distinguish it virtually from the anemone pulsatilla, a medicinal variety, of valued modern curative use, as a simple. This grows mostly in chalky soils which contain iron, and its blossoms are of a violet-blue colour. The active chemical principles of each plant are anemonin and anemonic acid. Probably the name pulsatilla is a diminutive of the Latin *puls.*, a pottage, as made from pulse, and used at sacrificial feasts. The appellation anemone signifies the wind-flower, from the Greek *anemos*, the wind. Pliny said this flower never opens but when the wind is blowing. It is also called pasque flower, from its blossoming at Easter, pask, or pass-over time. Each plant belongs to the same natural order, Ranunculaceæ. A tincture is made with spirit of wine from the entire plant, collected when in flower. This tincture is remarkably beneficial in disorders of the mucous membranes, alike of the respiratory and of the digestive passages. For catarrhal troubles of the eyes and ears, as well as for catarrhal diarrhœa, it is very serviceable; also for difficulties of menstruation in the female, and its use is always safe. As a medicine, it best suits persons of a quiet, gentle disposition, and of a lymphatic constitution, especially women; it is less appropriate for quick, active, energetic men. For indigestion, following a heavy or rich meal, pulsatilla tincture is almost a specific remedy. Three or four drops of this should be given at once, with a tablespoonful of cold water; and the same dose may be repeated after an hour, if then still needed. The flower of our wood-anemone tells the approach of night, or of a shower, by curling over its petals like a tent; and it has been said that fairies nestle within the tent, having first pulled its curtains round them. Among the old Romans, to gather the first anemone of the year was deemed a preservative against fever. The flower was placed in a scarlet cloth and kept undisturbed, unless the gatherer became ill, when it was tied round the neck or under the arm of the sufferer. Turner wrote of the anemone in 1568, "It may be called in English rose parsley, because there groweth a flower like a single rose in the middle of this herbe, which is very like persely in the leaves which lie about the root." The seed of the anemone, being very light and downy, is blown away by the first breeze of wind. A ready-witted French senator took advantage of this fact some years ago whilst visiting a covetous florist in the neighbourhood of Paris, who had long held a secret monopoly of certain very handsome and richly-coloured anemone plants from the East. Vexed to see one man hoard up for himself what ought to be more widely distributed, he walked and talked with the florist in the garden when the anemones were in seed. While thus occupied he let his robe fall upon the plants as if by accident, and so swept off a number of the little feathery seeds, which stuck fast to his garment, and which he afterwards cultivated when at home.

#### Angelica.

In the days of ancient Britain certain youths, bright of aspect, with light curly hair, blue eyes, fresh cheeks, and well-knit limbs, being carried as captives to Rome, were taken before Gregory the Great. He asked eagerly who they were, and being told English—Angli—he declared them to be not Angli, but Angeli—angels. So the herb angelica, brought to this country from northern latitudes in 1568 and now thoroughly naturalised in English gardens, owes its name, not to the land of its adoption, but to its reputed angelic healing virtues.

> "Angelica, the happy counter-bane,
> Sent down from heaven by some celestial scout,
> As well its name, and nature both avowt."

It belongs to the umbelliferous order of plants, and the cultivated medicinal variety of the herb, used as a simple, is called archangelica, because of its pre-eminently curative qualities. Besides occurring in our gardens it is grown abundantly near London, in moist fields, for the use of confectioners. Its candied aromatic stems are well known to most young persons as a favourite sweetmeat of the shops. They make an excellent stomachic, and relieve flatulence promptly. The roots of the garden angelica contain plentifully a peculiar resin called angelicin, which is stimulating to the secretions of the lungs and skin. An infusion of the plant may be made by pouring a pint of boiling water on an ounce of the bruised root, and a tablespoonful of this may be given three or four times in the day, or the powdered root may be given in doses of from ten to thirty grains. Likewise a dose of the dried seeds is ten grains. Angelica is beneficial in chronic rheumatism and gout. It undoubtedly possesses aromatic and invigorating qualities; whilst it is said also to cause a disgust for all spirituous liquors. In High Dutch it is named the root of the Holy Ghost. "This root," says Gerard, "is a singular remedy against all infections taken by corrupt, evil aire; if you dooe but take a piece of the root, and hold it in your mouth, or chew the same between your teeth, it doth most certainly drive away the pestilent aire." The roots should be dug in the autumn, and kept dry. Some have said that the archangelica was revealed by an angel, in a dream, to cure the plague. Others avow that it blossoms on the archangel St. Michael's day, May 8 (old style), and is, therefore, a preservative against evil spirits and witchcraft. The name archangel is also applied to the white, yellow, and red deadnettles.

#### Aniseed.

The aniseed—*pimpinella anisum*, of the umbelliferous natural order, is grown as a herb only seldom in our gardens, and is scarcely common enough to rank as an ordinary domestic simple; nevertheless, it possesses such useful aromatic virtues that a careful housewife will do well always to have some of the seed, closely bottled, in her store cupboard. The prefix, pimpinella, has been derived from the Latin *bipenella*, because of its secondary, featherlike leaflets. Aniseed contains three per cent. of a particular volatile oil, together with phosphates, malates, gum, and resin. The volatile oil, by its chemical basis, anethol, represents the medicinal properties of the plant. This oil has a special influence on the bronchial tubes to modify their secretions, and to encourage expectoration, particularly with children. When an attack of infantile catarrh has got beyond its first acute stage, aniseed-tea may be given with much advantage. It should be made by pouring half a pint of boiling water on three teaspoonfuls of the seeds, bruised in a mortar, and given, when cool, in doses of one, two, or three teaspoonfuls, according to the age of the child. For this reason the aniseed has been made an ingredient of both the English and the Scotch official paregoric tinctures to assist in the relief of chronic bronchitis. Furthermore, aniseed-tea and the essence of anise are excellent for relieving flatulent stomach-ache, whether in children or in adults. The essence is made with one part of oil to four parts of spirit of wine, and from ten to fifteen drops may be given on a lump of sugar for a dose. An old epithet of the anise was *solamen intestinorum*, a comforter of the bowels. Another old maxim says *oleum anisi prœsentaneum est venenum columbis*, "The oil of anise is a quick poison to pigeons." It likewise destroys lice and the itch insect, for which purposes it may be used in the form of an ointment. The leaves, if applied externally, will remove freckles. Gerard says: "The seed helpeth the yeoxing, or hicket [hiccough]; and should be given to young children to eat which are like to have the falling sickness; or to such as have it by patrimony, or succession. If it be taken with bitter almonds, it doth helpe the old cough." For the nightmare, and restlessness of languid digestion, a dose of essence of aniseed in hot water at bedtime is much commended. An odd literary mistake has been sometimes made in regarding anise as a plural noun. Thus we read in "The Englishman's Doctor," "Some anny seeds be sweet, and some more bitter."

